# Increasing Pediatric Palliative Care Consultation for Patients with Heart Disease and Prolonged Cardiac Intensive Care Stay

**DOI:** 10.1097/pq9.0000000000000796

**Published:** 2025-02-19

**Authors:** Andrew Headrick, Sarah Wawrzynski, Judson Moore, Melissa Winder, Jasmine R. Masih, Michele De Leon Jauregui, Brian Flaherty, Benjamin Moresco, Morgan M. Millar, Rachel R. Codden, Dominic Moore, Claudia Delgado-Corcoran

**Affiliations:** From the * Department of Pediatrics, Division of Cardiology, University of Utah at Primary Children’s Hospital, Salt Lake City, Utah; † Center for Healthcare Delivery Science, Nemours Children’s Health, Wilmington, De.; ‡ Division of Pediatric Palliative Care, Department of Pediatrics, University of Utah School of Medicine, Salt Lake City, Utah; § Division of Pediatric Critical Care Medicine, University of Utah, Salt Lake City, Utah; ¶ Division of Epidemiology, University of Utah, Salt Lake City, Utah

## Abstract

**Introduction:**

Patients with congenital heart disease are medically complex and experience high rates of morbidity and mortality. Pediatric palliative care (PPC) supports families navigating complex medical scenarios. This study evaluates the effect of quality improvement (QI) interventions towards increasing PPC consultations in patients with heart disease and cardiac intensive care unit (CICU) length of stay (LOS) ≥ 14 days.

**Methods:**

We conducted a mixed methods QI study using CICU team members and family survey assessments of PPC involvement. Patients with CICU LOS ≥14 days were eligible and comprised our study cohort. Interventions included the implementation of a digital prompt screening for eligible patients sent to CICU and PPC providers, as well as the implementation of weekly huddles to discuss consulted and eligible patients. Through the pre- and postintervention phases, family members of consulted patients and CICU team members were surveyed.

**Results:**

Preintervention (January 2020 to December 2021), PPC consultation rates were 35% (n = 34) and increased to 63% (n = 43) postintervention (January 2022 to February 2023) (*P* < 0.01). The timing of consultation was similar between phases. Survey results from family members (n = 19, 39% of 49 participants) and CICU team members (n = 80, 40% of eligible) revealed predominantly positive perceptions regarding PPC involvement.

**Conclusions:**

QI interventions, including collaborative huddles and digital prompts based on discrete screening criteria, lead to significantly increased rates of PPC consultation in patients with heart disease in the CICU. Survey results support PPC consultation as a positive contribution for families and CICU team members.

## INTRODUCTION

Patients with congenital heart disease (CHD) are medically complex and experience high rates of morbidity and mortality.^1^ Early pediatric palliative care (PPC) consultation for patients with CHD is an important but underutilized strategy to improve family experience and quality of care.^2,3^ PPC supports medically complex patients and their families in defining goals of care and facilitating advance care planning conversations.^2–9^ Families of pediatric patients facing life-threatening illnesses who receive PPC consultation report improved patient quality of life.^6^

To advocate that pediatric patients benefit from PPC, the Center to Advance Palliative Care (CAPC) published criteria in 2009 to identify critically ill pediatric patients who may benefit from PPC, including those with CHD.^10,11^ Furthermore, the American Academy of Pediatrics and Improving Palliative Care in the Intensive Care Unit Advisory Board expressed support for integrating PPC for critically ill patients as a standard of high-quality care.^12,13^ Despite efforts to integrate PPC into the care of patients with CHD, PPC consultations remain infrequent. When consultation occurs, it is typically late in a patient’s course in the context of high medical complexity and clinical decline, when patients and families may not reap the full benefit of PPC services.^2,9,14–18^

In the assessment of our institution’s use of PPC consultation, we previously reported low consultation rates for patients with CHD in our Cardiac Intensive Care Unit (CICU). In patients in our institution meeting the CAPC criteria of either a CICU LOS ≥14 days or at least 3 CICU admissions within 6 months, PPC consultation rates were 17%.^9^ Additionally, our group previously evaluated potential facilitators, barriers, and strategies to integrate PPC consultation for critically ill patients receiving mechanical circulatory support.^14^ This preceding work motivated this quality improvement (QI), providing insight into potential barriers and strategies to improve collaboration between the CICU and PPC teams.

The global aim of this project was to improve PPC consultation rates for patients cared for in the CICU with high medical complexity, as identified by a consecutive CICU length of stay (LOS) ≥14 days. Our specific, measurable, achievable, relevant, and time-Bound goal was to increase the rate of PPC consultation in patients with CICU LOS ≥14 days to at least 50% within 1 year of intervention. As additional measures, we evaluated the perceptions of patients’ families and CICU providers toward PPC involvement.

## METHODS

We utilized the Model for Improvement in this QI project.^19^ The institutional review boards at the University of Utah and Primary Children’s Hospital (IRB no. 00154513) approved this project. This manuscript was prepared in accordance with the SQUIRE 2.0 (Standards for Quality Improvement Reporting Excellence) guidelines.^20^

### Study Setting and Context

The CICU at Primary Children’s Hospital is a 16-bed unit with 500–600 total admissions annually. The institutional PPC team includes pediatric hospice and palliative medicine physicians, nurse practitioners, social workers, registered nurses, a chaplain, and mental health specialists. Our standard practice in the CICU before this project was to consult PPC at the discretion of the attending CICU physician, which led to a wide variety of consultation practices. Once consulted, the PPC team remains involved longitudinally until the condition has resolved, the patient transitions to adult medical care, or the patient dies.

Our multidisciplinary QI team included CICU providers, pediatric cardiologists, QI specialists, nursing, and palliative care physicians.

### Study Participants and Inclusion/Exclusion Criteria

Eligible patients were 0–21 years of age with at least 1 CICU admission with a consecutive CICU LOS ≥14. The preintervention phase included patients admitted from January 1, 2020, to December 31, 2021, and the postintervention phase from January 1, 2022, to February 28, 2023. We excluded patients if they received a PPC consultation before CICU admission.

### Interventions

**Fig. 1.**
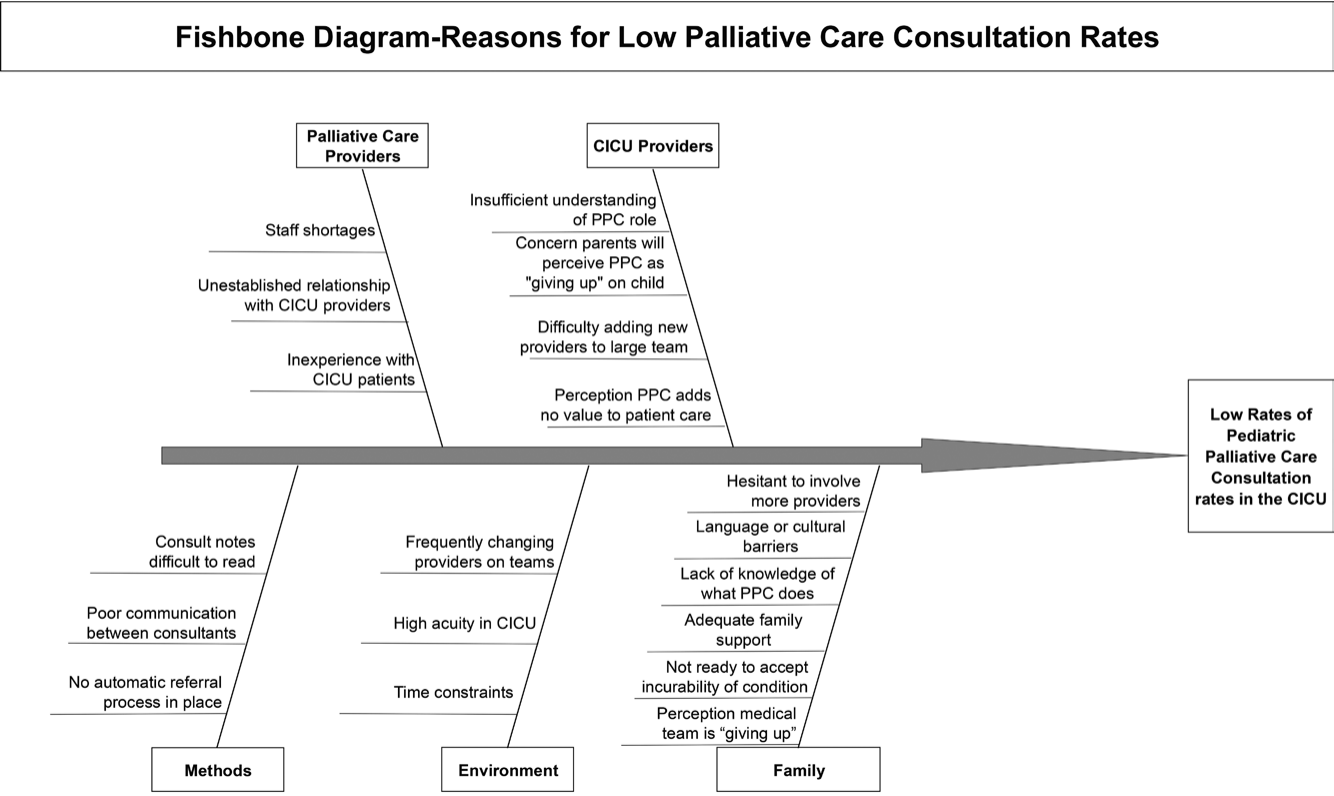
Fishbone diagram of factors influencing low PPC consultation rates in patients with congenital heart disease.

**Fig. 2.**
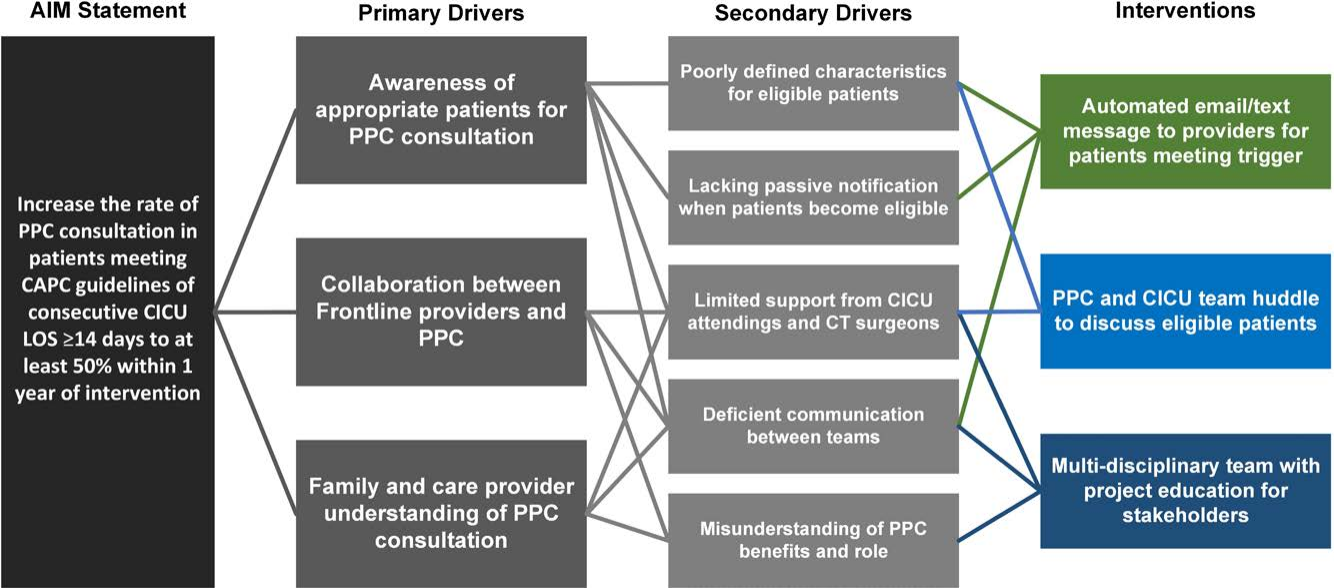
Key driver diagram for increasing PPC consultation in children with prolonged CICU hospitalization. CT, cardiothoracic.

Previous work investigating ICU providers’ barriers and facilitators toward PPC consultation identified that ICU providers valued PPC involvement in identifying families’ goals of care and decision-making but had reservations about the timing of consultation, interdisciplinary communication, and whether families may perceive PPC involvement as “giving up.”^2,14,21^ Our QI team met to review these data and discuss additional potential causes of low PPC consultation rates in patients meeting the CAPC guidelines and summarized these in a Fishbone diagram (Fig. 1). The 2 major issues we identified and sought to address were a lack of awareness from CICU providers of patients who could benefit from PPC consultation and confusion over the goals of PPC team involvement. The team mapped drivers and identified potential solutions using a key driver diagram (Fig. 2). In advance of implementation, the multidisciplinary QI team presented these proposed interventions to the PPC team, CICU physician, and nursing leadership in December 2021 to gain stakeholder support.

As a key driver included increasing the identification of eligible patients with CICU LOS ≥14 days, we first focused on developing an “automated digital prompt” that would alert the CICU provider that a patient’s CICU LOS was at 14 days. We worked with our hospital’s IT team to establish a weekly automated query of the CICU census identifying such patients and triggering an automated email to providers on service in the CICU. Given concerns about alert fatigue, we elected to send the email once weekly (Mondays). The email included a brief survey for CICU attending physicians on service to report reasons for nonconsultation. With our system of once-weekly census queries to identify patients, we recognized patients may not be identified promptly. To mitigate delays in PPC consultation, concurrent with the prompt, we implemented a weekly huddle between the CICU and PPC providers on Mondays. The huddle discussed current consultations and eligible patients and, as a contingency, to identify those approaching eligibility.

While piloting the automated digital prompt, we recognized that providers on service in the CICU or PPC may not see the email immediately. We also regularly noted patients that the automated query failed to identify. To adapt, the senior author manually reviewed the weekly list of eligible patients and sent the prompt via protected text message to the CICU and PPC providers on service, including the survey for nonconsultation.

### Data Collection

We collected data from the institutional heart center and Pediatric Cardiac Critical Care Consortium (PC4) databases.^22^ Data included demographics, heart disease diagnosis category (structural CHD, cardiomyopathy, or other [primary pulmonary hypertension or arrhythmia]), timing of diagnosis, hospital LOS, CICU LOS, timing of PPC consultation, mortality, and details surrounding deaths. Patients were identified as receiving a PPC consultation by the presence of a consultation note in their chart. We defined the timing of consultation as days from CICU admission to the first consultation note in the chart. Mortality data included number of deaths, age at death, mode of death, location of death, and time lapsed from PPC consultation to death.

### Measures

#### Outcome Measures

We defined our primary outcome measure as the rate of PPC consultation in patients with CICU LOS ≥14 days. As secondary outcome measures, we assessed, via survey, perceptions of patients, families, and CICU team members (CICU physicians, advanced practice providers, and nurses) toward PPC role and involvement. Families of patients receiving consultation during the study period (pre- and postintervention) were contacted and consented to surveys conducted either in-person, by mail, or by email. CICU team members received surveys via email during the postintervention phase, with consent inferred by voluntary participation.

#### Balancing Measures

We monitored the time to PPC consultation to assess if the identification of patients with CICU LOS ≥14 caused unintended delays in consultation. Additionally, we evaluated the total number of PPC consults in the CICU during both phases to assess PPC team workload.

#### Process Measures

We assessed the accuracy and frequency of the digital prompt the senior author sent via email, whether automated or manually. Similarly, we evaluated the frequency of the scheduled weekly huddle on Monday. The senior author reviewed both weekly.

### Statistical Analysis

Demographic and clinical characteristics are reported using descriptive statistics. Continuous values are reported as median with interquartile range (IQR). We made comparisons of demographics and categorical outcomes using the Pearson chi-square test or Fisher exact test, and continuous variables with Wilcoxon rank-sum test. A *P* value of <0.05 was considered statistically significant. To assess changes in PPC consultation rates, we utilized a P-chart with all patients with CICU LOS ≥14 days as the denominator and those with PPC consultation as the numerator. Given variability in the number of eligible patients by month, we grouped consultation rate observations into 2-month blocks to create each point on the p-chart. We use data from January 2020 to December 2021 to establish a baseline centerline, +/1 δ, ±2δ, and upper and lower control limits.^23^ We applied standard process control chart rules. We analyzed all data using JMP, version 17 (SAS Institute, Inc., Cary, N.C., 1989–2023).

## RESULTS

### Demographics

Over the study period, 1,630 admissions to the CICU had a median CICU LOS of 3 days. In the preintervention phase, there were 945 total CICU admissions, with 112 (12%) patients who experienced a CICU LOS ≥14 days; of these, we excluded 15 due to prior PPC consultation, 2 of which occurred prenatally. In the postintervention phase, there were 685 total CICU admissions, with 85 (12%) patients who experienced a CICU LOS ≥14 days; of these, we excluded 17 due to prior PPC consultation, 10 of which occurred prenatally. Table 1 summarizes the demographic and clinical characteristics of the pre- and postintervention groups. Apart from distribution of heart disease category (*P* = 0.02), there were no significant differences between the groups.

**Table 1.** Clinical Characteristics and Demographics of Patients with CICU LOS ≥14 Days in the Pre- and Postintervention Phases, Stratified by PPC Consultation, or None

Variable, n and (%) or median [IQR]	All Patients with CICU LOS ≥ 14			No PPC Consultation			PPC Consulted		
	Preintervention	Postintervention	*P*	Preintervention	Postintervention	*P*	Preintervention	Postintervention	*P*
N	97	68	—	63	25	<0.01*	34	43	<0.01*
Female	34 (35)	29 (43)	0.33*	20 (32)	10 (40)	0.46*	14 (41)	19 (44)	0.82*
Age at CICU admission, d	2 [0–92]	10 [0–157]	0.37†	1 [0–56]	1 [0–66]	0.98†	15 [0–173]	16 [0–206]	0.79†
Prenatal diagnosis	59 (61)	37 (54)	0.70*	37 (59)	13 (52)	0.51*	22 (65)	24 (56)	0.26*
Heart disease category			0.02‡			0.25‡			0.02‡
Structural	88 (91)	58 (85)		61 (97)	22 (88)		27 (79)	36 (84)	
Cardiomyopathy	8 (8)	3 (5)		1 (2)	1 (4)		7 (21)	2 (5)	
Other	1 (1)	7 (10)		1 (2)	2 (8)		0 (0)	5 (12)	
CICU LOS, d	23 [16–36]	22 [16–38]	0.71†	20 [16–27]	18 [15–20]	0.04†	40 [19–67]	32 [21–53]	0.56†
Hospital LOS	35 [27–62]	38 [26–74]	0.61†	31 [27–42]	28 [23–37]	0.08†	68 [30–136]	48 [34–110]	0.66†
CICU admission to PPC, d	—	—	—	—	—	—	10 [6–24]	13 [6–21]	0.98†

Clinical characteristics and demographics of patients with prolonged CICU LOS in the pre- and postintervention phases, stratified by PPC consultation or none.

* Chi-square test.

† Wilcoxon rank sum.

‡ Fisher exact test.

### Mortality

Mortality data are summarized in Table 2. There were 35 deaths during the study period in patients with CICU LOS ≥14 days, with similar incidence in the pre- and postintervention phases. PPC consultation as significantly more common (*P* < 0.01) in patients that experienced mortality (n = 18, 23.4%) versus those who received no consultation (n = 2, 2.3%). Most patients died in the CICU, with greater than half dying after discontinuation of life-sustaining therapies. The mode and location of death did not vary significantly across phases. Though not significant, PPC consultation was earlier in the postintervention phase, with time from consultation to death increasing from a median 15 to 98 days.

**Table 2.** Mortality Data and Outcomes in Patients with CICU LOS ≥ 14 days

Variable, n and (%) or median [IQR]	All Patients with CICU LOS ≥ 14			No PPC Consultation			PPC Consulted		
	Preintervention, n = 97	Postintervention, n = 68	*P*	Preintervention, n = 63	Postintervention, n = 25	*P*	Preintervention, n = 34	Postintervention, n = 43	*P*
Mortality	20 (21)	15 (22)	0.82*	4 (6)	1 (4)	1.0†	16 (47)	14 (33)	0.24*
Age at death (d)	134 [52– 297]	175 [79–336]	0.58‡	337 [40–468]	77 [77–77]	0.32‡	127 [52–231]	182 [94–350]	0.21‡
Mode of death			1.00*						1.00*
Withdrawal of life-sustaining therapies	11 (55)	9 (60)		1 (25)	1 (100)	0.57†	10 (63)	8 (57)	0.86†
Other (unsuccessful CPR, brain death, comfort)	9 (45)	6 (40)		3 (75)	0 (0)		6 (37)	6 (43)	
Location of death			0.12†			0.52†			0.10*
CICU	13 (65)	14 (93)		3 (80)	1 (100)		10 (63)	13 (93)	
Out of hospital	5 (25)	0 (0)		1 (20)	0 (0)		4 (25)	0 (0)	
In-hospital or ED, not CICU	2 (10)	1 (7)		0 (0)	0 (0)		2 (12)	1 (7)	
Time from PPC consultation to death (d)	—	—		—	—	—	15 [8–101]	98 [23–168]	0.14‡

CPR, cardiopulmonary resuscitation; ED, emergency department.

* Chi-square test.

† Fisher exact test.

‡ Wilcoxon rank sum.

### Outcome Measures

**Fig. 3.**
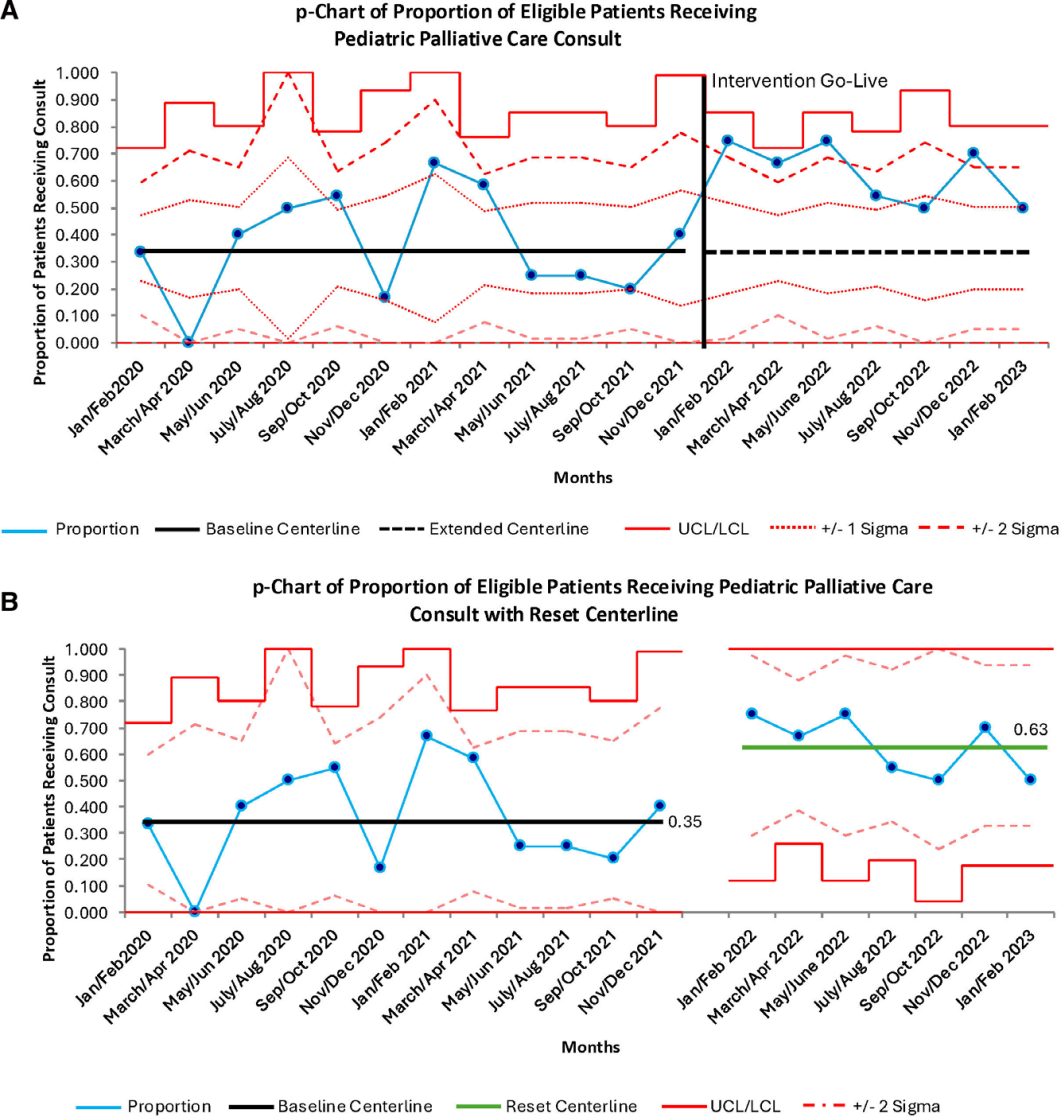
p-Charts depicting the proportion of eligible patients receiving a PPC Consult. A, Containing a centerline, upper and lower control limits, ±2, and ±1δ lines calculated from baseline data (January/February 2020–November/December 2021). Special cause variation is noted with the first 2 postintervention points (January/February and March/April 2022) falling beyond 2δ, the first 4 postintervention points (January/February 2022–July/August 2022) falling beyond 1δ, and 8 consecutive points starting in November/December of 2021 falling above the centerline. B, The p-chart with reset centerline, control limits, ±2δ, and ±1δ lines. The reset portion is calculated from data from January/February 2022 (when special cause variation was first noted in the postintervention period) through January/February 2023.

**Fig. 4.**
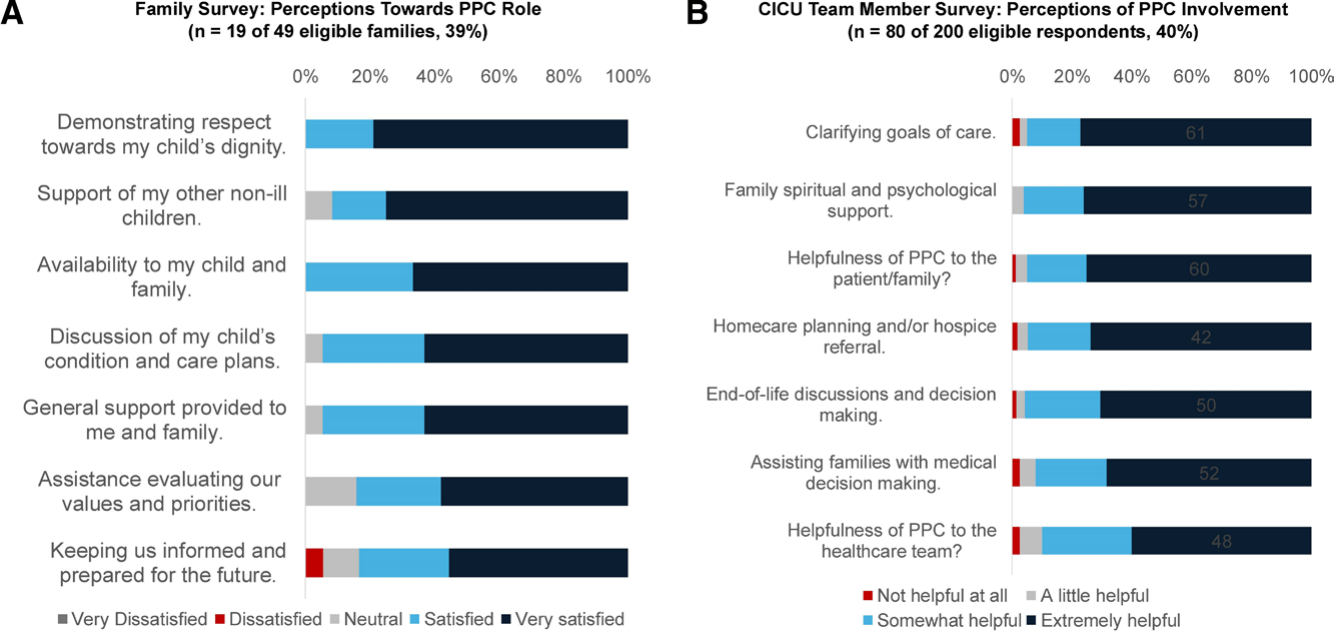
Stacked bar graphs of results from (A) family survey and (B) CICU team member survey..

1. PPC consultation frequency: The p-chart summarizes rates of PPC consultation over the study phases (Fig. 3). Figure 3A displays all data points with a baseline centerline,±1δ,± 2δ, and upper and lower control limits calculated from data obtained from January 2020 to December 2021. Multiple indications of special cause variation are noted in Figure 3A: (1) the first 2 postintervention points (January/February and March/April 2022) fall beyond 2δ, (2) the first 4 postintervention points (January/February 2022–July/August 2022) fall beyond 1δ, and (3) 8 consecutive points starting in November/December 2021 fall above the centerline (Fig. 3A). Although the November/December 2021 data are included in the baseline preintervention period, this time point includes our initial education efforts for the consultation prompt and weekly huddle, and the increase in consultation rate seen in this point may represent the initial effects of our PPC consult education. Regarding sustainability, all points in the postintervention period representing 14 months of data are above the baseline center line. To demonstrate the change in process, Figure 3B depicts the p-chart with a reset centerline utilizing data from January/February 2022 (when special cause was first noted in the postintervention period) through January/February 2023. As an additional method to confirm differences in PPC consultation rates between the pre- and postintervention periods, we compared the preintervention rate (34 of 97, 35%) to the postintervention rate (43 of 68, 63%) with a chi-square test. We noted a significant difference (*P* < 0.01).
2. Family survey, rates, and reasons for family PPC refusal: Of the 77 patients that received PPC consultation during the study period, 49 families were approached, and 19 completed the survey (response rate = 39%) assessing perceptions toward PPC involvement (Fig. 4A). Satisfaction (response of “very satisfied” or “satisfied”) was most present in: “the respect the palliative care team demonstrated towards my child’s dignity” (100%), “availability of the palliative care team to my child and family” (100%), and “the way the palliative care team discussed my child’s condition and plans of care” (95%).
A majority of respondents (n = 14, 79%) indicated that when the PPC team became involved, they did not perceive that it was “primarily because [their] child was dying.” Similarly, a majority (n = 13, 68%) responded “not applicable” to the question of whether they perceived the purpose of PPC involvement was to facilitate end-of-life discussions. In the postintervention phase, two families refused PPC consultation (3.4%), with reported motives as “unknown” and the presence of “enough family support at home.”
3. CICU team member survey: A total 80 of 200 eligible respondents completed a survey (response rate = 40%) assessing perceptions toward PPC involvement in the CICU (Fig. 4B). Respondents consisted of 50 bedside nurses (62.5%), 16 CICU physicians (20.1%), and 14 advanced practice providers (17.5%). The 3 aspects of PPC involvement most consistently rated as helpful (response of “extremely helpful” or “somewhat helpful”) and were “clarifying goals of care” (95%), “providing family spiritual and psychological support” (96%), and “homecare planning and/or hospice referral” (95%). Areas where respondents most identified PPC consultation as “not helpful” or only “a little helpful” included “provider spiritual and psychologic support” (29%) and “assisting in symptoms management” (27%).
4. Attending CICU physician refusal to PPC consultation: Of 68 eligible patients, attending physicians refused consultation in 25 (36.8%) cases, citing that the patient is doing well, the ICU provider felt comfortable addressing palliative care needs, or that involvement of PPC may negatively influence families’ decision-making.

### Balance Measures

1. Timing of PPC consultation postintervention: Timing from CICU admission to PPC consultation did not differ significantly between the pre- (median 10, IQR 6–24 d) and postintervention (median 13, IQR 6–21 d) groups (*P* = 0.98).
2. Impact of additional consultation on the PPC team workload: Among all patients hospitalized in the CICU, the average annual new PPC consultations increased from 24 in the preintervention phase to 70 postintervention.

### Process Measures

1. Frequency of automated digital prompt: At the outset of the postintervention phase, the automated census query did not consistently identify all patients with CICU LOS ≥14 days. This was noted in a manual review by the senior author, with the automated query identifying eligible patients 50% of the time. Despite several iterations, the coding issues persisted. The senior author began manually reviewing the census and sending the prompt via email and text. This continued through the postintervention phase, with CICU and PPC team members remarking on their preference toward the text message format. The manual process took place all but 3 weeks throughout the postintervention phase.
2. Frequency of the PPC and CICU team weekly huddle: Aside from holidays, the huddles occurred every week through the postintervention phase.

## DISCUSSION

This pre- and postintervention QI study successfully achieved the SMART goal without negatively impacting the timing of consultation but with a notable increase in the systematic workload of the PPC team. Importantly, patients’ families and CICU team members find PPC consultation beneficial. Finally, we report several challenges that can inform similar future projects in designing automated digital prompts to consultation.

PPC consultation rates more than doubled in the postintervention period and coincided with the implementation of prompts alerting CICU providers that a PPC consult was appropriate and weekly huddles between the CICU and PPC consult teams. Apart from the heart disease category, pre- and postintervention demographics were similar, and no other interventions or systematic changes were undertaken during the study period. Although this change in distribution reflected increased admissions for primary pulmonary hypertension or arrhythmias, we have no reason to expect this institutional variation year-over-year in heart diagnosis affected PPC consultation rates. Thus, the observed increase in PPC consultation is reasonably attributable to implementing our QI interventions.

Families surveyed in our study reported that PPC consultation assisted in evaluating and conveying their values and priorities. Notably, most respondents did not identify PPC consultation as being associated with their child dying or facilitating end-of-life discussions. This is consistent with previous literature that families appreciate PPC involvement earlier in their child’s disease trajectory to discuss advance care planning and symptom management rather than during acute deterioration or death.^24^

CICU team member survey responses revealed perceptions of PPC collaboration that mirrored those of existing literature.^14,21,25,26^ Barriers to PPC consultation cited included the perception that the patient is doing well, the primary provider felt comfortable addressing palliative care needs, and PPC involvement may negatively influence families’ decision-making. Compared with family survey results, this reflects similar previously described differences in perception between families and CICU providers regarding the role of PPC, the timing of consultation, and the benefits of the longitudinal care offered.^27,28^ Importantly, these discrepancies in patient family and provider perceptions can inform future interventions to increase PPC consultation in the CICU. Furthermore, education on the nature and goals of PPC, the services rendered, and increased collaboration may beget shifts in ICU provider perception over time.

As a part of balancing measures in this project, we considered whether implementation of the primary intervention might lead to consultation occurring later in the admission. However, this was not evident in our cohort, which had a similar timing of consultation relative to CICU admission in the pre-and postintervention groups. Furthermore, in the postintervention phase, variability in timing of consultation decreased. In planning, we predicted that PPC workload would increase with an estimate of 1 new consultation per week. New CICU PPC consultations slightly exceeded these estimations. Following the study period, the PPC team responded to this increased demand, dividing the service into 2 teams, which were distributed by diagnosis and hiring new providers. Implementing such interventions requires thorough and thoughtful consideration of available resources, given the potential for significant demand increases. The success of the current QI approach creates opportunities for further targeted QI interventions to improve rates of PPC consultation in patients meeting other CAPC recommendations, including those with underlying genetic disorders and complex CHD.

Finally, the automated query was challenging, frequently failing to identify eligible patients, necessitating manual monitoring. Fortunately, manual screening of the electronic medical record was easily incorporated. Thus, this is a generalizable strategy to retrieve eligible patients without requiring coding to automate the process.

## LIMITATIONS

This was a single-center study and is subject to inherent limitations. Second, the need for manual census review for eligible patients may limit the application of this practice, though a successful automated process would lead to a similar result. Third, CICU team member survey responses were not differentiated by professional role, and the low response rate among staff members may be limited in capturing the full range of opinions and experiences. Finally, family surveys lacked demographic data on race, ethnicity, or spoken language, which may fail to elucidate recognized inequities that exist in pediatric critical care.^29^

## CONCLUSIONS

This is the first work evaluating the effectiveness of QI interventions toward increasing PPC consultation rates in patients with CHD in the CICU and builds on previous successes applying similar interventions in other pediatric populations.^14,15,30^ CICU LOS ≥14 days created a framework for providers to reduce the variability in when and to whom the consultations occurred and overcome referral biases. Family and CICU team surveys informed changes for improvement and sustainability of PPC service delivery. The project demonstrated the power of collaboration among stakeholders, improving care that benefited patients and families with prolonged CICU LOS.

## ACKNOWLEDGMENTS

The author acknowledge the Primary Children’s Pediatric Palliative Care interdisciplinary team who helped with the consultations and Jennifer Sweatman for assistance with Pediatric Palliative Care data retrieval.
